# Crystal structure of alkyl hydroperoxidase D like protein PA0269 from *Pseudomonas aeruginosa*: Homology of the AhpD-like structural family

**DOI:** 10.1186/1472-6807-11-27

**Published:** 2011-05-26

**Authors:** Teresa E Clarke, Vladimir Romanov, Yuri N Chirgadze, Chananat Klomsiri, Gera Kisselman, Jean Wu-Brown, Leslie B Poole, Emil F Pai, Nickolay Y Chirgadze

**Affiliations:** 1 Campbell Cancer Research Institute, Ontario Cancer Institute, Princess Margaret Hospital, University Health Network, Toronto, Ontario M5G 2C4, Canada; 2 Institute of Protein Research, Russian Academy of Sciences, 142292 Pushchino, Moscow Region, Russia; 3 Department of Biochemistry, Wake Forest School of Medicine, Winston-Salem, NC 27157, USA; 4 Departments of Biochemistry, Molecular Genetics, and Medical Biophysics, University of Toronto, Toronto, Ontario, Canada; 5 Department of Pharmacology and Toxicology, University of Toronto, Toronto, Ontario M5S 1A8, Canada

## Abstract

**Background:**

Alkyl hydroperoxidase activity provides an important antioxidant defense for bacterial cells. The catalytic mechanism requires two peroxidases, AhpC and AhpD, where AhpD plays the role of an essential adaptor protein.

**Results:**

The crystal structure of a putative AhpD from *Pseudomonas aeruginosa *has been determined at 1.9 Å. The protein has an all-helical fold with a chain topology similar to a known AhpD from *Mycobacterium tuberculosis *despite a low overall sequence identity of 9%. A conserved two α-helical motif responsible for function is present in both. However, in the *P. aeruginosa *protein, helices H3, H4 of this motif are located at the N-terminal part of the chain, while in *M. tuberculosis *AhpD, the corresponding helices H8, H9 are situated at the C-terminus. Residues 24-62 of the putative catalytic region of *P. aeruginosa *have a higher sequence identity of 33% where the functional activity is supplied by a proton relay system of five residues, Glu36, Cys48, Tyr50, Cys51, and His55, and one structural water molecule. A comparison of five other related hypothetical proteins from various species, assigned to the alkyl hydroperoxidase D-like protein family, shows they contain the same conserved structural motif and catalytic sequence Cys-X-X-Cys. We have shown that AhpD from *P. aeruginosa *exhibits a weak ability to reduce H_2_O_2 _as tested using a ferrous oxidation-xylenol orange (FOX) assay, and this activity is blocked by thiol alkylating reagents.

**Conclusion:**

Thus, this hypothetical protein was assigned to the AhpD-like protein family with peroxidase-related activity. The functional relationship of specific oligomeric structures of AhpD-like structural family is discussed.

## Background

Alkyl hydroperoxidases play an important role in detoxifying peroxides and other reactive oxygen species in the cell. As part of an effort to identify and characterize potential antimicrobial targets from the pathogenic microorganism *Pseudomonas aeruginosa*, the hypothetical protein PA0269 from the carboxymuconolactone decarboxylase (CMD) family was selected for structure determination. This protein family includes a few representative members: the first, carboxymuconolactone decarboxylase, is involved in biodegradation of monocyclic aromatic carbon sources, the second, alkyl hydroperoxidase, is related to protecting against oxidative stress and the third has an unknown function. Carboxymuconolactone decarboxylase catalyzes decarboxylation of γ-carboxymuconolactone to β-ketoadipate enol-lactone in the catabolism of aromatic compounds through the protocatechuate branch of the β-ketoadipate pathway (EC 4.1.1.44). Known structures for this family include the trimer *Mycobacterium tuberculosis *AhpD [1-3], the hexameric protein TTHA0727 from *Thermus thermophilus *[4] and some additional hexameric hypothetical proteins from thermophilic and methanobacteria from structural genomics consortium efforts.

Important antioxidant protection is provided by the AhpC/AhpD system, where in *M. tuberculosis*, the proteins have no sequence identity but work under the same promoter and can oligomerize [5,6]. Mycobacterial peroxiredoxin alkyl hydroperoxide reductase C (AhpC) is a member of the family of non-heme peroxidases which protects heterologous bacterial and human cells against oxidative and nitrosative injuries [7]. AhpC metabolizes peroxides a conserved N-terminal cysteine residue, which undergoes oxidation [8]. At the final step of the catalytic cycle, the cysteine residue must be reduced. AhpD has been shown to play a role as a component of the AhpC/AhpD-dependent system since the mycobacterial lysate supports peroxidase activity of AhpC only in the presence of AhpD [1]. Thus, alkyl hydroperoxidase D is an adaptor protein completing the AhpC/AhpD-dependent system, exhibits peroxidase activity and is involved in the antioxidant defense mechanism.

The mechanism of activity of alkyl hydroperoxidase D is mostly characterized for AhpD from *M. tuberculosis*. In the thioredoxin-like active site of *M. tuberculosis *AhpD, the catalytic residues Cys130 and Cys133 within the Cys-Ser-His-Cys sequence are responsible for peroxidase activity [1-3]. The proposed reaction mechanism of AhpD is based on a proton relay system consisting of five residues, Glu118, Cys130, His132, Cys133 and His137, and one structural water molecule [2]. This mechanism was strongly supported by site-directed mutagenesis experiments and enzyme kinetics [3]. The arrangement of the active site residues of the other CMD family member, carboxymuconolactone decarboxylase, have not been determined. In the *Thermus thermophilus *TTHA0727 structure, the analogue of the essential catalytic residue Cys130 residue of AhpD is replaced with a corresponding Ser70, indicating that this protein lacks peroxidase activity its function is unknown [4].

Here, we report the crystal structure and putative functional analysis of the hypothetical protein PA0269 from the pathogenic bacteria *P. aeruginosa *at 1.9Å resolution. Despite low overall sequence identity to *M. tuberculosis *AhpD, two catalytic sulfhydryl groups of cysteine residues and other functionally important residues around these cysteines form an exact configuration of the proton relay system with a very similar active site. Further examination has revealed the proteins share a conserved structural motif containing all the residues of the active site in a hairpin of two α-helices. This suggests the hypothetical *P. aeruginosa *protein may function as a weak hydroperoxidase, as verified by the ability to reduce H_2_O_2 _in the FOX assay, and possibly as the adaptor protein AhpD in an AhpC/AhpD-dependent peroxidase system. Using the homology of the conservative hairpin motif, we have found five additional related protein structures with yet unknown or uncharacterized functions. These proteins were also assigned to the AhpD-like protein family with probable peroxidase activity.

## Results

### Protomer protein structure

**Figure 1:**
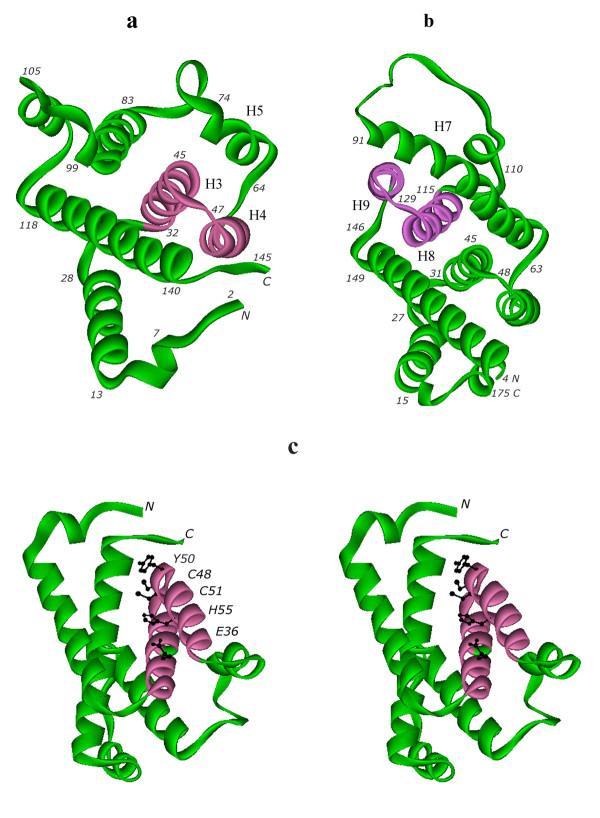
**Crystal structures of the hypothetical protein PA0269 from (a) *P. aeruginosa *and (b) alkyl hydroperoxidase D (AhpD) from *M. tuberculosis ***[2]. Although the proteins have similar function, the topologies of the proteins differ. Note the conserved helix motif (pink) has a different position in each structure. (c) Stereoview of the putative alkyl hydroperoxidase D from *P. aeruginosa*. The five side chains of the residues of the active site involved in the proton relay system are shown as black atoms.

The crystal structure of a single molecule of PA0269 is presented in Figure 1a (PDB code 2O4D). It belongs to class of globin-like α-proteins and consists of ten helical fragments ranging in size from 4 to 25 residues. Four Se-Met residues were found in the structure with the Se in partial occupancy. It is important to compare this protein structure with the structure of the functionally well characterized *M. tuberculosis *AhpD presented in Figure 1b (PDB code 1GU9). The proteins from *P. aeruginosa *and *M. tuberculosis *have different chain lengths: 144 and 177 amino acid residues, respectively, but both consist of ten helical segments. These structures have very low sequence homology with a residue identity for the whole chain of only 9.0%. The protein from *P. aeruginosa *has five helices H2, H3, H4, H7, and H10 ranging from 15 to 25 amino acid residues, while AhpD from *M. tuberculosis *has seven such helices H3, H4, H5, H7, H8, H9, and H10 ranging from 12 to 29 residues lengths of helices, for example, H5 (*P. aeruginosa *residues 65-72, Figure 1a), and H7 (*M. tuberculosis *residues 64-91, Figure 1b) differ by more than three fold.

However, in a particular section of the protein chain containing the key catalytic Cys residues, spanning residues 24-62 of PA0269 and residues 107-145 of *M. tuberculosis *AhpD, the sequence identity increases to 33%. This section of sequence corresponds to a conserved motif of two α-helices, H3 (residues 31-47) and H4 (residues 48-62) in the *P. aeruginosa *protein structure and H8 (residues 113-129) and H9 (residues 130-144) in the *M. tuberculosis *AhpD structure. The hairpin of the conserved two-helical motif is highlighted in somewhat different orientations in the two proteins in Figure 1. The configurations of two helices H3 and H4 for *P. aeruginosa *and corresponding H8 and H9 for *M. tuberculosis *structures are similar. However, they are localized at different sections of the protein chain. This might explain why the pair wise sequence identity of these proteins is extremely low. This also suggests that comparing the protein chains by the superposition of C_α _atoms would be very difficult and the rmsd-value of C_α _atoms cannot be reasonably assessed.

The transposition of the two conserved helix hairpin motifs along the protein chain is an interesting feature. In fact, the sequence motif created by H3, H4 occurs in the N-terminus in the *P. aeruginosa *structure but the equivalent H8, H9 helices are found at the C-terminus of *M. tuberculosis *AhpD. The genes coding the two proteins with similar function appear to have been mutated during the evolutionary process. It is important to note that the general all-helical motif of the protein tertiary structures is retained in both.

### Establishment of the putative active site of PA0269

A stereoscopic view of the structure of PA0269 is presented in Figure 1c. The possible catalytic residues located in the sequence fragment Cys-Ala-Tyr-Cys were compared with the fragment Cys-Ser-His-Cys in the sequence of *M. tuberculosis *AhpD. The putative catalytic residues Cys48 and Cys51 of the *P. aeruginosa *protein are located in helix H4. All five putative catalytic residues of the proton relay system, Glu36, Cys48, Tyr50, Cys51 and His55, are located in two adjacent helices, H3 and H4 at the N-terminus of the protein chain, while in *M. tuberculosis *AhpD, the five catalytic residues found in the helices H8 and H9 at C-terminus of the protein chain as Glu118, Cys130, His132, Cys133 and His137.

Only one substitution in the series of catalytic residues, *P. aeruginosa *His132 corresponds in position to Tyr 50 in *M. tuberculosis*. This may have some weakening effect on the catalytic peroxidase activity of the *P. aeruginosa *protein mainly due to a difference in pKa of the residues. This value for tyrosine is approximately 10.1, while for the histidine side chain it is about 6.9. However, these values could vary, depending on the local environment of residues inside the protein molecule. In addition, the *M. tuberculosis *residue His132 (in the position corresponding to *P. aeruginosa *Tyr50) is not absolutely conserved in AhpD from different species, indicating that its catalytic role could be played by alternative residues [2].

**Figure 2:**
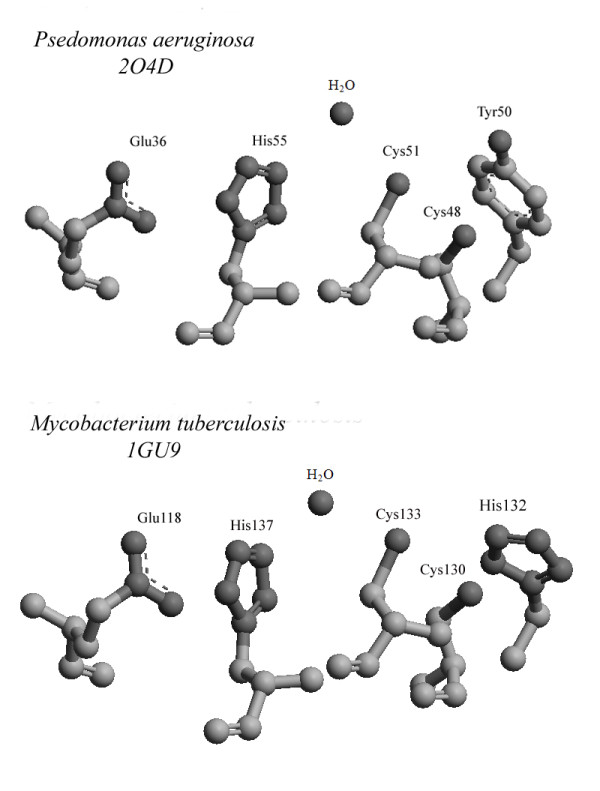
**Amino acid residues of the active sites of putative alkyl hydroperoxidase D from *P. aeruginosa *and from *M. tuberculosis ***[2]. The arrangements of the residues and water molecule involved in the proton relay system mechanism are shown.

The arrangement of the residues involved in the proton relay system mechanism is shown for these two protein structures in Figure 2. The biochemical reaction mechanism also includes a unique molecule of structural water (located 3Å from the nearest residue His55 in PA0269), which is present in both proteins. The detailed arrangements of the residues and water molecule in both proteins are very similar, allowing us to propose that the putative active site of PA0269 confers peroxidase activity. Direct confirmation of the activity of PA0269 using the existing assay format for *M. tuberculosis *AhpD would be difficult and requires the specific partner proteins [3]. However, the structural similarity strongly suggests that the protein PA0269 from *P. aeruginosa *may have function similar to alkyl hydroperoxidase D. At the moment, the binding site of the substrate has not been determined. In the monomer of AhpD from *P. aeruginosa *and *M. tuberculosis*, the substrate could bind near the catalytic cysteines accessible on the external surface of the protein.

### Testing the ability to reduce hydrogen peroxide

**Figure 3:**
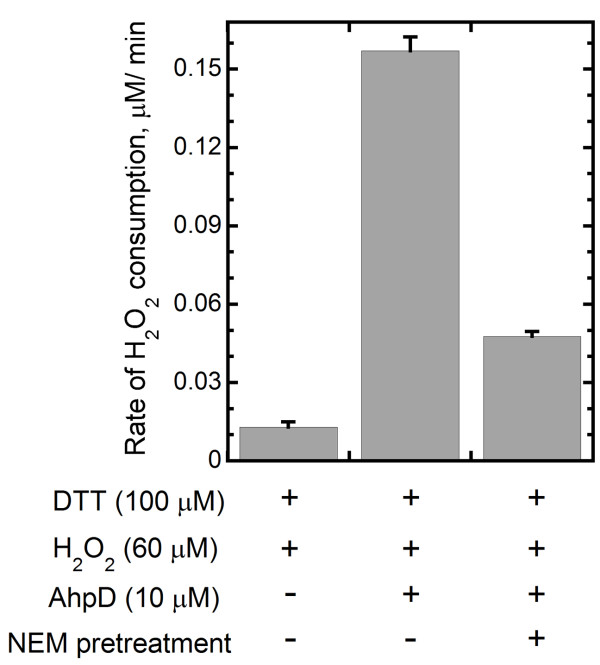
***P. aeruginosa *AhpD shows cysteine-dependent peroxidase activity as determined by the FOX assay**. Initial rates are the average of three experiments. In one set of experiments, reduced AhpD was preincubated with 80 mM NEM (N-ethylmaleimide) to alkylate free thiols in AhpD prior to the FOX assay.

The ability of *P. aeruginosa *AhpD (PA0269) to reduce H_2_O_2 _was tested using the FOX assay. *P. aeruginosa *AhpD showed peroxidase activity at a rate of 0.16 ± 0.01 μM H_2_O_2_/min under assay conditions (Figure 3). Thus, this protein from *P. aeruginosa *exhibits a weak peroxidase activity, similar in function to the previously characterized AhpD protein from *M. tuberculosis*.

Interestingly, the activity is strongly inhibited by preincubation of the reduced protein with N-ethylmaleimide, confirming that the peroxidase activity is cysteine dependent (Figure 3).

### Structural homology of peroxidase-related AhpD-like protein family

Using the sequence of PA0269 from *P. aeruginosa *as a query, a set of homologous proteins with known structure was examined. Fourteen structures from the Protein Data Bank were identified by using the protein BLAST-server with a sequence score from 286 to 25 bits and a sequence residue identity from 100% to 9%. The first related structure was the same protein but crystallized in a different crystal form, space group C2, deposited in the PDB with code 2IJC[9]. All other related protein structures have a lower residue identity from 42 to 9%. Among them, five structures from this list were selected provided the sequences contained the possible catalytic motif of Cys-X-X-Cys and their atomic structures were publically released in the PDB. All of these proteins are presented in Table 1 but only the crystal structure of *M. tuberculosis *AhpD has been characterized and described in a publication [2]. The second protein of unknown function, 2GMY, has 42% residue identity. Three other uncharacterized peroxidase-related proteins with PDB codes 2PRR, 2OYO, 2OUW, and protein 1GU9 have rather low sequence identity values of 24, 24, 9, and 9%, respectively. In these structures, residue similarities are found only in a limited part of the sequence. For example, the homology with AhpD from *M. tuberculosis *is observed only for residues 24-62, as numbered for the protein with PDB code 2O4D. The overall lengths of the protein chains are varied, from 144 to 197 amino acid residues.

**Table 1:** Sequence homology of PDB structures of the putative alkyl hydroperoxidase D-like structural family as compared with the *P. aeruginosa *structure (PDB code 2O4D)

PDB code	Description of proteins and species	Residue identity	Residue positives*
2GMY	Protein of unknown function from *Agrobacterium tumefaciens*, putative antioxidant, defense protein AhpD Length, 153 residues	42.4% (61/144)	64.6% (93/144)
2PRR	Alkyl hydroperoxidase AhpD core protein uncharacterized peroxidase-related from *Ralstonia eutropha *Length, 194 residues	23.6% (34/144)	37.5% (54/144)
2OYO	Uncharacterized peroxidase-related protein from *Deinococcus geothermalis *Length, 196 residues	23.6% (34/144)	30.6% (47/144)
2OUW	Alkyl hydroperoxidase AhpD core protein from *Rhodospirillum rubrum *Length, 138 residues	9.0% (13/144)	11.1% (16/144)
1GU9	Alkyl peroxidase AhpD from *Mycobacterium tuberculosis *Length, 177 residues	9.0% (13/144)	13.9% (20/144)

Note. Residue positive (*) means similarity with polar-polar or nonpolar-nonpolar pair residues.

Protein structure with PDB code 1GU9 was published in ref. [2]. Atomic coordinates of all other structures do not include publications and their PDB accession codes are given in the first column.

**Figure 4:**
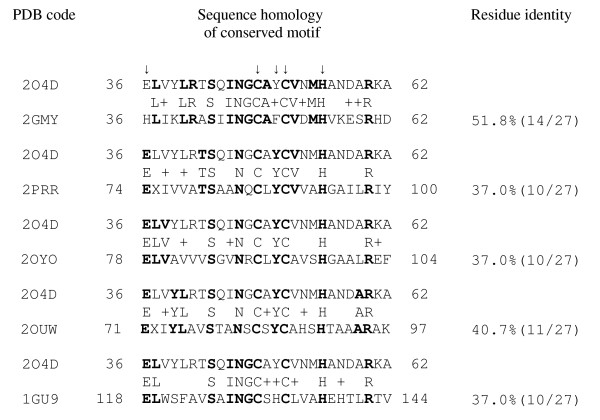
**Sequence homology with the *P. aeruginosa *structure (PDB code 2O4D) of the conserved α-helical hairpins responsible for putative peroxidase-related functional activity.** Notes: 1. Chain fragments are pair compared with the range of 36-62 positions of AhpD from *P. aeruginosa *(PDB code 2O4D) as query. Identical residues are marked as bold ones. 2. Arrows in the upper line indicate the residues involved in reaction mechanism. 3. Residue positive (+) means similarity with approximately polar-polar and nonpolar-nonpolar pair of residues.

The most interesting parts of the chains are the conserved structure of the two-helical hairpin containing the putative catalytic residues. The sequence homology to the motif responsible for function, helices H3 and H4 of *P. aeruginosa *structure, is provided in Figure 4. A high similarity within this residue range is observed only for the protein with PDB code 2GMY. Surprisingly, in the other proteins, the conserved motif is situated in a different place of the chain, being shifted from N-terminal to the C-terminal end. The sequence identity varies from 52 to 37% and the number of positive similar residues (i.e. similarity mainly with polar-polar or nonpolar-nonpolar residue pair) differs from 70 to 44%, respectively.

**Figure 5:**
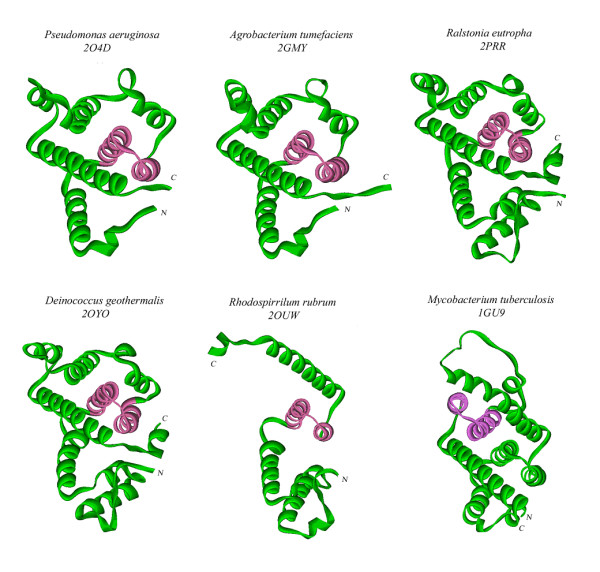
**Protein chain topologies of *P. aeruginosa *AhpD and several known structures of the alkyl hydroperoxidase D-like protein family**. The functionally important cores of two α-helical segments conserved in all the structures are marked with pink color in each.

It is not typical to analyze the overall structural similarity combined with such a small level of sequence identity (less than 30%). To demonstrate the similarity, we have presented all the protein structures in the same projection with the conserved motif colored in each (Figure 5). The protein fold of the structure with PDB code 2GMY is very similar in details despite rather low residue identity 42.4%. The proteins with PDB codes 2OYO and 2PRR have extra N-terminal and C-terminal helices and even less residue identity, 23.6% in both cases. Despite this, they retain similar protein topologies and location of conserved motifs. Using the DALI server, the rmsd of 2O4D with 2GMY is 1.2 Å, 2OYO is 1.8 Å, and 2PRR is 2.1 Å. The situation is different for the protein with PDB code 2OUW, where a significant part of structure disappears. However, the conserved helical hairpin is located at about the same place, retaining their peroxidase-related features. The next unusual case is realized in the structure of 1GU9 (Figure 5). Here, the topologies of proteins with PDB codes 2O4D and 1GU9 are very similar. As previously noted, the conserved motif H3, H4 of 2O4D appears in the protein 1GU9 at a different location than H8, H9, closer to the C-terminal end of the chain (see Figure 1a). The main feature of this peroxidase-related protein family appears to be the presence of a conserved functional motif of the α-helical hairpin rather than the specific topology of the protein molecule. Since all five residues involved in the functional process are located inside the conserved motif, the active sites of protomer proteins are always isolated from the rest of the structure. That means they are "structurally independent" as it was initially suggested [2]. This important feature seems to be inherent for all related members of this peroxidase-related protein family as shown in Figure 5.

**Figure 6:**
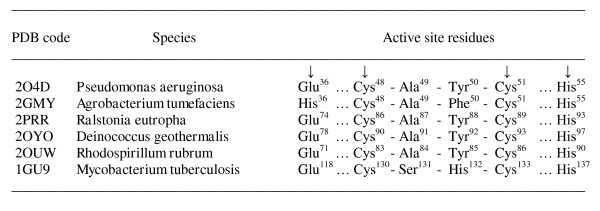
**Sequence homology of residues involved in the reaction mechanism for the identified proteins of the alkyl hydroperoxidase D-like structural family**. *Arrows indicate the residues involved in the reaction mechanism.

The sequence homology of the residues involved in the proton relay system of these proteins is shown in Figure 6. Of the four important residues located in positions 36, 48, 51 and 55 (numbering as in *P. aeruginosa *structure, code 2O4D), residues Cys48, Cys51, and His55 are invariant for all members of protein family. These three residues are the most essential ones for performing a catalytic reaction [3]. Glu36 is substituted by His in AhpD from *Agrobacterium tumefaciens *(2GMY) but a histidine residue could also play the role as a deprotonating agent instead of a glutamic acid. Other substitutions are Ala49-Tyr50 by Ser131-His132 in *M. tuberculosis *AhpD (1GU9), located in the spacer region between two catalytic cysteines. Residue Tyr50 is present in all members with exception of Phe50 (2GMY) and His132 (1GU9). It was suggested the tyrosine could alternatively play a catalytic role in different AhpDs [3] but a histidine in this position present in sequences of some other species.

The crystal structure of protein TTHA0727 from *Thermus thermophilus *is also partially similar to the structure of AhpD-like proteins. The study of this structure shows the existence of a γ-CMD related structural family with various functional proteins [4]. This protein also has sequence homology to the AhpD-like protein from *Moorella thermoacetica*, hypothetical protein AF0348 from *Archaeoglobus fulgidus *and AhpD-like protein from *Mezorhizobium sp. *with pair wise sequence identity of 35%, 29% and 23%, respectively [4]. The superposition of three other known structures revealed a conserved motif of three α-helices numbered as H4, H5, H6 in TTHA07027 where helices H4, H5 can be compared with H3, H4 in the AhpD-like protein PA0269 from *P. aeruginosa*. In spite of the similarity in topologies, the AhpD-like protein family and γ-CMD protein family need to be considered as two different families. In fact, the sequence identity motifs are very different in the three helices of the γ-CMD family compared to the two helices of AhpD-like family. It is also significant that the five catalytic residues of the proton relay system responsible for peroxidase activity or the entire catalytic sequence motif Cys-X-X-Cys are not found in these members γ-CMD family members.

### Oligomeric structure of alkyl hydroperoxidases D in crystal

**Figure 7:**
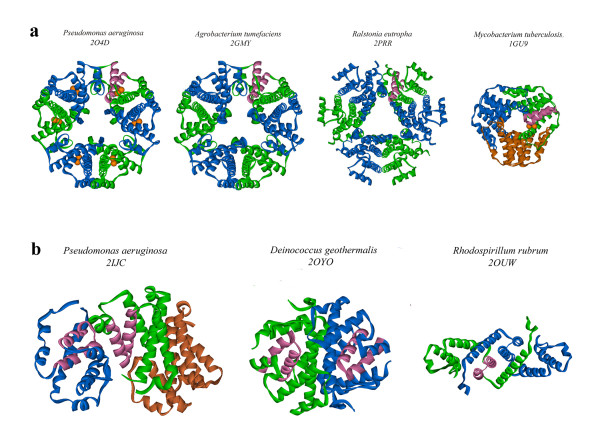
**(a) Examples of hexamers and trimer molecular units found in the crystal for alkyl hydroperoxidase D-like proteins from different species; (b) Examples of trimer and dimer complexes observed in the crystal of alkyl hydroperoxidase D-like proteins for some species**. (a)In all cases, the three-fold symmetry rotation axis is crossing the figure plane in the center of the oligomer complexes. One functionally important conserved motif is marked by pink color in each structure and the two catalytic cysteine sulfhydryl groups are shown as orange balls. (b)The trimer has antiparallel packing of subunits in the crystal structure. Proteins which occur as dimers are related by a two-fold symmetry rotation axis crossing the figure plane in the center of complexes. The functionally important conserved motif is marked by pink color. The bacterial species origin and PDB structure codes are shown at the top.

Crystals of *P. aeruginosa *AhpD belong to space group P6_3_22 (code 2O4D), where the asymmetric unit contains one protein molecule. However, in the crystal structure, the protomer molecule forms many contacts with other molecules. As a result, an oligomer is observed as a planar hexameric ring as shown in Figure 7a. In relation to the 2-fold crystallographic symmetry rotation axis in the plane of this ring, two protomer molecules are arranged in a dimer by hydrophobic interactions and hydrogen bonds. Further, the whole hexamer is formed by means of crystallographic symmetry rotation along the 6_3_-axis perpendicular to the ring plane and crossing its center. This oligomeric state is consistent with the gel filtration data indicating an apparent molecular weight of 102 kDa, or calculated oligomeric state of 5.5 (data not shown). Thus, the biological unit of AhpD from *P. aeruginosa *might be a hexamer with an external diameter of approximately 49 Å, thickness of about 18 Å, and size of the central hole of about 13 Å, as measured with C_α _backbone model. It is important to note that the conserved motif and two catalytic cysteines are structurally independent and accessible for each molecule in the oligomer, as seen in the figure.

The situation is slightly changed when *P. aeruginosa *AhpD is crystallized in a different space group, C2 (code 2IJC, ref. [9]). Here, the asymmetric unit contains two oligomers, with a total of 9 protein chains. The asymmetric unit content can be easily divided in two independent oligomers. One oligomer is a hexamer with the shape of a plane hexameric ring and it is exactly the same as described above for the structure 2O4D, while the second oligomer is a trimer, forming an entity with an antiparallel arrangement of associated protein subunits, as shown in Figure 7b.

If a set of similar oligomeric complexes from different species are compared, some complexes have the form of planar rings with a central holes and three-fold rotation symmetry axis as presented in Figure 7a. Complexes of different types are displayed in Figure 7b. Here, examples of asymmetric trimer and symmetrical dimers with two-fold rotation symmetry axis are shown. It should be noted that for the proteins of two structures, 2OYO and 2OUW, the similar hexameric forms also have been observed in the crystalline state. The authors of these structures state the hexameric form is the functioning biological unit, supported by the observation of oligomers in solution. However, in all cases, it is confirmed that the conserved portion of the protein responsible for function is structurally independent of the oligomeric state.

## Discussion

The crystal structure of the hypothetical PA2069 protein allows the recognition of a putative catalytic helix-turn-helix motif containing the sequence Cys-X-X-Cys. Structural analysis shows the conserved arrangement of several side groups of adjacent residues which could be necessary for constructing a proton relay system involving a single molecule of structural water. Unfortunately, in all protein members of the AhpD-like family, the binding sites of possible substrates or inhibitors are not yet known, although a small cavity on the surface of protein near the catalytic cysteines is available.

We have analyzed the crystal structures of a total of six proteins with putative or uncharacterized peroxidase activity. They have rather low sequence identity with the exception of approximately 30-residue spans containing the catalytic cysteines. Nonetheless, all proteins have a common conserved motif of two α-helices which include five residues of the proton relay system. This allows us to suggest the functional activity of the proteins is similar to AhpD. Analysis of the functional assignment of PA0269 was done by comparison with the functionally characterized *M. tuberculosis *AhpD structure. The organization of *ahpD *and *ahpC *genes in *M. tuberculosis *and *ahpD, ahpC *and *ahpF *genes in *P. aeruginosa *are different. In *M. tuberculosis*, the *ahpD *and *ahpC *genes are located in one operon with some other genes of metabolic proteins (GenBank accession NZ_ADAB 01000068). In *P. aeruginosa*, the gene for PA0269 (*ahpD*) is located in one operon while the genes for PA0139 (*ahpC*) and PA0140 (*ahpF*) are located in another operon (GenBank accession NC_002516). This difference could be one of the reasons for weak peroxidase activity which we have observed in the simplified biochemical assay.

An analysis of the AhpD-like family homology shows that the structure and sequence of the conserved domain are very similar but the remainder of each protein varies significantly. We could consider such a phenomenon as a case of molecular convergent evolution while maintaining the functional activity of the peroxidase as the leading factor. The conservation of activity is provided by retaining the conserved motif of two hairpin α-helices. Among the members of the AhpD-like family, we have observed an unusual situation where the conserved motif in the *M. tuberculosis *AhpD structure belongs to a topologically distinct region of the protein compared to *P. aeruginosa *AhpD and other members of family. It means this particular protein is built with a different folding pattern and different topology than all other members of the AhpD-like family. Even though all members of the protein family belong to one class of globin-like α-proteins, different chain topologies are allowed. A transposition of part of the gene sequence is possible as long as the peroxidase activity is retained. Maintaining the common structural class of an all-helical fold is very favorable because it seriously simplifies the folding mechanism and increases the speed of evolution, as in the case of *M. tuberculosis *AhpD.

A variety of oligomeric forms has been observed between the AhpD-like protein family members. Most often they exist in a hexamer form, although they may appear as a trimer or dimer. Large contact areas between pairs of adjacent molecules suggest that oligomeric complexes could occur not only in a crystalline state but also in solution. For several proteins, this is observed experimentally, allowing some authors to define the biological unit as an oligomer. However, the biologically relevant assembly may be influenced by the presence of AhpC in the cell. Nevertheless, the functionally important conserved two-helical motif is always structurally independent which suggests that the peroxidase activity is retained both for separate monomers and their oligomers.

## Conclusion

Recent progress in expressing recombinant proteins and producing Se-methionine isomorphous crystals, as well as successes in various methods of protein crystallography, have resulted in a growing of number of protein structure files deposited in the Protein Data Bank. Structural homology can be used successfully to classify proteins to protein families only when sequence residue identity is higher than 30-35% along the majority of the protein chain. Also, a number of structures with putative or unknown function are likely related to a known protein family but have limited sequence homology and are not fully functionally characterized [10]. In this communication, a similar problem to identify the functional activity of related protein structures has been resolved by searching for specific conserved structural motifs responsible for the functional activity. As a result a total of five uncharacterized proteins from various species have been assigned to the AhpD-like protein family.

## Methods

### Cloning, Expression and Purification

The target gene PA0269 (encoding the full length protein of 145 aa) was amplified from *P. aeruginosa *genomic DNA (strain 633, ATCC# 17933D) with the forward primer 5'-GCGGCGGCCCATATGACCACCCGCCTCGAATG-3' and the reverse primer 5'-GCGCGGATCCTTATCATTCCGGTTGCATGCCC-3' and inserted into a pET15b vector (Novagen, USA) using standard methods. Protein for crystallization experiments was expressed in *E. coli *BL21 (DE3) cells using the M9 selenomethionine high-yield media kit (Medicilon, CA, USA), harvested and flash-frozen. Expression of native protein for the assay was carried out in Terrific Broth using the same host. Cells were thawed and lysed by sonication (Branson, VWR) on ice in the presence of 0.5% CHAPS, 0.2 mM PMSF, 0.5 mM benzamidine and 300 units of benzonase (Novagen, WI) in binding buffer (50 mM HEPES pH 7.5, 0.5 M NaCl, 5 mM imidazole and 5% glycerol). The mixture was clarified at ~69 000 g (24 000 rpm in a Beckman J-25I, JA-25.50 rotor) for 40 min and applied to a DE52 column (Whatman, UK) pre-equilibrated with binding buffer. The flow-through was applied to a column of Ni-NTA Superflow (Qiagen, USA), washed with binding buffer followed by wash buffer (binding buffer with 0.03 M imidazole) and elution buffer (binding buffer with 0.25 M imidazole). The fractions containing the protein were pooled and concentrated. The selenomethionine sample and half of the native sample were immediately subjected to gel filtration. The remainder of the native sample was treated with thrombin to cleave the polyhistidine tag and passed through a Ni-NTA column to remove uncleaved protein prior to size exclusion chromatography. Gel filtration of all samples was performed on a Superdex 200 26/60 column (GE Healthcare, USA) equilibrated with gel filtration buffer (10 mM HEPES pH 7.5, 0.25 M NaCl, 0.5 mM TCEP). Fractions of the pure protein were pooled and concentrated to 94 mg/ml (SeMet sample) or 50 mg/ml (native protein sample with cleaved His tag). The protein was flash-frozen and stored at -80°C. MALDI-ToF mass spectrometric analysis of tryptic fragments confirmed the identity of the protein and incorporation of SeMet.

### Protein crystallization

The selenomethionine labeled protein was thawed and diluted to 15 mg/ml with gel filtration buffer. The protein was centrifuged at 14 000 rpm for 10 minutes at 20°C. Crystals were obtained after a few days by sitting drop vapor diffusion against a reservoir solution containing 0.1 M Tris at pH 7.6, 0.2 M sodium chloride, 0.4 M sodium dihydrogen phosphate and 1.6 M dipotassium hydrogen phosphate in CrystalClear strips (Hampton Research, CA, USA) at 20°C. Crystals were flash-frozen in a mixture of reservoir and 20% ethylene glycol.

### X-ray data collection and structure determination

Diffraction data were collected at 100K using the Advanced Photon Source beamline 17-ID, Argonne National Laboratory. SAD X-ray data to 1.85 Å resolution were processed with the HKL2000 suite [11] and phases were determined with the program autoSHARP [12]. Automatic model building successfully built and docked 139 of 145 residues of the molecule in the asymmetric part of crystal unit cell. The structure was refined against the SAD dataset with the CCP4 program suite [13] and rebuilt using COOT [14]. The electron density for the protein main chain was continuous and only the first Met and His-tag were not modeled. Molprobity was used to analyze the quality of the model [15]. Final data and refinement statistics can be found in Table 2. The atomic coordinates have been deposited in the RCSB Protein Data Bank with the accession code 2O4D.

**Table 2:** Summary of X-ray Data and Structure Statistics

*Crystallographic Data:*	
Space group	P6_3_22
Unit cell dimensions (Å)	92.72, 92.72, 65.30
Resolution (Å)	50-1.85 (1.92-1.85)*
Wavelength (Å)	0.979337
Observations (unique reflections)	14,576
Multiplicity	21.5 (20.2)
<I/σ(I) >	28.5 (9.7)
R_merge _(%)	6.2 (40.2)
Completeness (%)	99.9 (100)
* *Numbers in parentheses are for the highest resolution shell.*	
	
*Refinement and structure statistics:*	
R_work _(%)	17.6
R_free _(%)	20.6
RMS Deviations from ideal geometry	
Bond length (Å)	0.011
Bond angle (°)	1.219
Number of atoms	
Protein non-hydrogen atoms	1,140
Water oxygen atoms	108
	
Mean B-factor (Å^2^) for protein atoms	28.9
Mean B-factor (Å^2^) for water	37.3
Ramachandran plot statistics (%)	
Residues in most favoured regions	96.1
Residues in allowed regions	3.9
PDB ID	2O4D

### The ferrous oxidation-xylenol orange (FOX) assay

Ten μM of reduced protein and 100 μM DTT were mixed in 50 mM potassium phosphate buffer pH 7.0 at room temperature. In one set of experiments, reduced protein was preincubated with 80 mM NEM (N-ethylmaleimide) to alkylate free thiols in the protein prior to the FOX assay. The reaction was initiated by the addition of 60 μM hydrogen peroxide (H_2_O_2_). The concentration of remaining hydrogen peroxide was determined at various time points up to 120 min by a xylenol orange-iron reaction. Briefly, 35 μl of the reaction was removed from the reaction mixture and added to 665 μl of FOX reagent (250 μM ammonium ferrous sulfate, 125 μM xylenol orange, 100 mM sorbitol, and 25 mM sulfuric acid). The FOX mixture was incubated for at least 20 min at room temperature and the absorbance at 560 nm was then monitored. Residual H_2_O_2 _concentration in the reaction was calculated using a standard curve. The assay was performed in triplicate.

### Structural homology search

A structural similarity search was carried out using the DALI server [16]. Protein secondary structure comparison was performed with the use of the service at the European Bioinformatics Institute [17]. Sequence alignment and homology analysis were performed with the program suites protein BLAST [18] and advanced search PDB from the Protein Data Bank [19]. Three-dimensional protein structures were superposed with Swiss PDB-Viewer [20]. PyMOL was used to examine the protein structures and present figures [21].

## Abbreviations

AhpD: alkyl hydroperoxidase D; CMD: carboxymuconolactone decarboxylase; FOX: ferrous oxidation-xylenol orange assay, used for detection hydrogen peroxide.

## Authors' contributions

TEC carried out crystal structure determination, performed structural data analysis, and drafted the manuscript. JWB and VR carried out protein production and purification for crystallization and functional studies experiments. GK performed protein crystallization. CK and LBP have performed functional studies and been involved in drafting the manuscript. YNC performed analysis of possible function and homology of the related protein family, and drafting the main part of manuscript. EFP carried out data analysis and has been involved in revising manuscript. NYC conceived of the study, and participated in its design and coordination. All authors read and approved the final manuscript.
